# Breathing patterns during sleep and their relationship with FEV1 in pediatric patients with cystic fibrosis residing at high altitude

**DOI:** 10.3389/fped.2024.1360227

**Published:** 2024-08-15

**Authors:** Elida Duenas-Meza, Nadia Juliana Proaños-Jurado, Sarah Pulido-Fentanes, Diego F. Severiche-Bueno, María Isabel Escamilla-Gil, Maria Angelica Bazurto-Zapata, Jenny Libeth Jurado, Miguel Ricardo Suarez, Luis Fernando Giraldo-Cadavid

**Affiliations:** ^1^ Departamento de investigación CINEUMO, Fundación Neumológica Colombiana, Bogotá, Colombia; ^2^ Facultad de Medicina, Universidad de La Sabana, Chía, Colombia

**Keywords:** cystic fibrosis, sleep, high altitude, obstructive sleep apnea, sleep disorder breathing (SDB)

## Abstract

**Introduction:**

Sleep-disordered breathing (SDB) and gas exchange disorders are common in patients with cystic fibrosis (CF). Currently, the impact of the disease on sleep patterns in patients living at high altitude and the relationship of these patterns to lung function are largely unknown. The aim of this study was to determine the frequency of SDB in children with CF aged 6–18 years and the relationship between SDB and lung function (FEV1).

**Methods:**

This is an analytical cross-sectional study of children aged 6–18 years diagnosed with CF. Spirometry before and after bronchodilators and polysomnography with capnography were performed. Descriptive analysis of qualitative and continuous variables was performed. Spearman's correlation coefficient was used to determine the correlation between polysomnogram and lung function (FEV1).

**Results:**

Twenty-four patients with CF were included. The mean age was 10.5 ± 3.1 years and 62.5% were male. Nine children had bronchiectasis on chest CT. The median absolute baseline FEV_1_ was 1,880 (1,355–2,325) ml and 98% (83%–110%) of predicted value. No significant difference in FEV_1_% was observed between subjects with obstructive sleep apnea (OSA) and those without OSA (*P* = 0.56). The prevalence of OSA was 66.7% in children younger than 13 years and 40% in children older than 13 years. The Spearman correlation coefficient between FEV_1_ and percentage of total sleep time with saturation less than 90% (T90) was rho −0.52 (*p*-value = 0.018), and between FEV1 and percentage of total sleep time with saturation less than 85% (T85) was statistically significant with rho −0.45 (*p*-value = 0.041). A positive correlation was observed between FEV_1_ and SpO_2_ during sleep with rho 0.53 and a statistically significant *p*-value (0.014).

**Conclusions:**

A high prevalence of sleep apnea was found in children with CF living at high altitude, with a negative correlation between FEV_1_ and T90 and T85 oxygenation indices, and a positive correlation between FEV_1_ and SpO2 during sleep.

## Introduction

Cystic fibrosis (CF) is a disease with a worldwide distribution, although the incidence, carrier rate, and type of mutation vary depending on the population and ethnic group analyzed. In Latin America, CF is estimated to affect between 1 in 1,600 and 1 in 14,000 live births (1). A pilot study conducted in Bogotá that included neonatal screening for CF found an incidence of 1 in 8,297 (2). According to the National CF Registry, 64% of CF patients in Colombia live at medium altitude (1,500–2,500 m) and 32% of patients live at high altitude (2,500–3,500 m) (3).

Clinical studies in adult CF patients have focused on findings at sea level and the tolerance of patients to altitude changes during air travel, analyzing the effects of these altitude changes on lung function, arterial oxygen pressure (PaO2) and clinical manifestations (4). CF patients can develop hypoxemia and hypercapnia during exercise and sleep (5). The drop in barometric oxygen pressure with altitude and subsequent hypoxemia leads to hyperventilation to restore PaO_2_, which in turn lowers PCO_2_. This process is driven by changes in central and peripheral chemoreceptor sensitivity, adjustments in cerebral blood flow, changes in pulmonary artery pressure, and adjustments in the macro- and micro-architecture of sleep. In addition, exposure to high altitude has been shown to produce an increase in apnea-hypopnea index (AHI), which is explained by an increase in centrally triggered events. However, as the acclimatization process progresses, the AHI begins to decrease (6). The study of arterial gases at high altitude at our institution showed that PCO_2_ and PO_2_ levels are lower than those described at sea level (7). In adult obstructive sleep apnea (OSA) patients, desaturation indices were found to be worse at high altitude than those reported at sea level (8). It is important to note, however, that all these studies have been conducted in adults and not in children.

Sleep-disordered breathing (SDB) is an underdiagnosed comorbidity in children with cystic fibrosis (CF). Nocturnal hypoxemia, OSA and nocturnal hypoventilation are common in patients with lung disease. These are known to have neurocognitive, cardiovascular, and quality of life consequences (9). A high prevalence of OSA in CF patients, independent of age and lung function, has been described in a recent meta-analysis (10). However, a correlation between disease severity assessed by FEV_1_ and nocturnal saturation has been described (11, 12).

To our knowledge, no studies have been conducted at high altitude to determine the effects of chronic hypoxia on the breathing patterns of children with CF during sleep. The aim of this study was to investigate the breathing patterns during sleep in children with CF living at high altitude and to correlate these patterns with lung function.

## Materials and methods

### Study subjects

Twenty-four children aged 6 to 18 years referred to the CF Center of the Fundación Neumológica Colombiana between January 2020 and December 2021 with a confirmed diagnosis of CF by iontophoresis and/or genetic study and who resided in a city at a high altitude (2,640 m) were included. Children who were unable to perform spirometry and children who had experienced an exacerbation of their disease within six weeks before entering the study were excluded. All participants had an informed consent form signed by a parent or guardian. Those over the age of 14 also signed an informed assent form in accordance with local regulations.

To detect a statistically significant difference in lung function (FEV1) between subjects with and without OSA, a sample size calculation was performed based on an expected mean difference of 14% with a standard deviation (SD) of 6.5%. This effect size was derived from a previous analysis of FEV1 in patients with cystic fibrosis (CF) aged 6–18 years treated at the Cystic Fibrosis Center in Fundación Neumológica Colombiana. Assuming a two-tailed alpha of 0.05, a power (beta) of 80%, a 1:1 allocation ratio and a parametric distribution, a sample size of 24 participants was determined. This sample size was sufficient to detect a significant correlation coefficient of at least 0.5 between FEV1 and AHI or oxygen desaturation index.

### Study design

This is an analytical cross-sectional study of children between 6 and 18 years of age diagnosed with CF and prospectively recruited at the CF Center of Fundación Neumológica Colombiana in Bogotá, Colombia, a city located at high altitude (2,640 m). The study was approved by the Ethics Committee. Registry Number: 2019006–24507.

### Polysomnogram

The polysomnogram (PSG) was performed according to the guidelines of the American Academy of Sleep Medicine (AASM) using commercially available digital equipment (Philips Respironics®; Alice 5 and LE models). It was performed at the Fundación Neumológica Colombiana Cystic Fibrosis Center by a PSG technologist with pediatric experience, in a dark and quiet room with an average room temperature of 19°C. The duration of the polysomnogram was 7–10 h. A parent or guardian was present throughout the study.

The following parameters were measured: chest wall and abdominal motion assessed by inductance plethysmography, heart rate assessed by electrocardiogram (ECG) (DII), and airflow monitored by nasal pressure cannula and oronasal thermistor. Arterial SpO2 values and heart rate during wakefulness and sleep were recorded with a high-precision pulse oximeter (Masimo; Rad 8 model) integrated into the digital PSG acquisition system with simultaneous pulse wave recording, as well as bilateral electroencephalograms (EEGs), three EEG channels (F4, C3, C4, O2), chin and anterior tibial electromyograms, and an analog output from a body position sensor.

Sleep architecture was assessed using standard techniques in accordance with AASM recommendations and guidelines (13–15). Sleep stages were classified as non-rapid eye movement (NREM) sleep and rapid eye movement (REM) sleep. The proportion of time spent in each sleep stage was expressed as a percentage of total sleep time (TST). Apnea index was defined as the number of apneic episodes per hour of TST.

The following definitions were used: (1) *obstructive apnea*: absence of airflow (90% reduction in airflow signal) with continuous chest and abdominal wall movements lasting at least two breaths; (2) *central apnea* (CA): a 90% reduction in airflow signal with absence of chest or abdominal movements lasting 20 s or at least two breaths and associated with 3% oxygen desaturation; (3) *mixed apnea*: an event that meets the criteria for apnea for at least two breaths and is associated with no respiratory effort during one part of the event and the presence of inspiratory effort during another part: An event that meets the criteria for apnea for at least two breaths and is associated with no inspiratory effort during part of the event and the presence of inspiratory effort during another part, regardless of which part comes first; (4) *Hypopnea*: A 30% decrease in nasal flow for two or more breaths with a corresponding decrease in SpO2 (3%), a microarousal, or both (14, 15).

Obstructive sleep apnea (OSA) was defined from a polysomnographic perspective as follows: In children younger than 13 years: Obstructive Apnea-Hypopnea Index (OAHI): ≥ 2/h with severity classified as mild (2–4.9/h), moderate (5–9.9/h), or severe (≥10/h) (16). Children 13 years of age and older: OAHI ≥5/h with severity classified as mild (5–14.9/h), moderate (15–29.9/h), or severe (≥30/h).

PSG oxygen saturation parameters include the following:

- Oxygen desaturation index (ODI): number of desaturations ≥3% per hour of total sleep time
- T90: percentage of total sleep time with saturation less than 90%.
- T85: percentage of total sleep time with saturation less than 85%.
- Minimum saturation (nadir SpO2): minimum saturation during apneas/hypopneas
- Average saturation during respiratory events: average saturation recorded during apneas and hypopneas.
- Desaturation: SpO2 value less than 90%.

### Spirometry

Spirometry was performed before and after bronchodilators according to the American Thoracic Society (ATS) guidelines. FEV1 was used as a functional parameter of severity. Spirometry was performed with a properly calibrated spirometer (Vyntus® SPIRO). All participants performed spirometry according to ATS guidelines (17).

Additional definitions include the following (18): *Normal spirometry:* Percentage values for FVC, FEV_1_ and peak flow (PEF) of 100 ± 20% of predicted and for %FEF _25–75_ 100 ± 35% of predicted, without response to bronchodilator *Abnormal spirometry*: Values below 80% for FVC, FEV_1_, %FEV_1_/FVC ratio and PEF and less than 70% for %FEF _25–75_ *Bronchodilator Response*: A change of >10% relative to the predicted value for FEV1 or FVC in accordance with the ATS/ERS technical standard (19).

### Statistical analysis

Qualitative variables were described as absolute and relative frequencies. For continuous variables, measures of central tendency and dispersion were used according to the assumption of normality evaluated by the Shapiro-Wilk test. Spearman's correlation coefficient was used for non-parametric samples to determine the correlation between polysomnogram parameters and lung function (FEV_1_). All hypothesis systems were tested two-tailed, and a value of *p* < 0.05 was considered statistically significant. Analyses were performed using Stata 16© statistical software.

## Results

A total of 24 children diagnosed with CF, living at high altitude and without exacerbations in the 6 weeks prior to recruitment were included. The mean age of the children was 10.5 ± 3.1 years, 72.2% were in the normal weight percentile and 62.5% (15/24) were male. The mean sweat chloride concentration was 103 ± 32.8 and 83.3% (20/24) were found to have digital clubbing.

Regarding the findings on chest computed tomography, 9 of 19 children had bronchiectasis and 23 of the patients had a sputum culture in the last trimester, of these 30.43% (7/23) showed colonization by *Staphylococcus aureus* and 4.34% (1/23) by *Pseudomonas aeruginosa*. The mean number of hospitalizations for pulmonary exacerbations in the previous year was 2.3 ± 2.6. It was found that 75% (18/24) of the children received pancreatic enzymes, 83% (20/24) vitamins, 70.8% (17/24) nutritional supplements and 79.2% (19/24) dornase alpha. None of the participants were on CFTR modulators (Table 1). Eight were p.F508del homozygous and 3 were compound for p.F508del mutation (Table 2).

**Table 1:** General characteristics of the population.

Variables	Total population *n*: 24
Sex, male	15 (62.5)
Age, years	10.5 ± 3.1
Percentiles	
Underwight	2 (8,3)
Normal wight	19 (79,2)
Overweight	2 (8,3)
Obesity	1 (4,2)
Genetic study	23 (95.8)
p.F508del Homozygous	8 (34.7)
Bronchiectasis in chest CT *n*:19	9 (50.0)
Presence of rales	2 (8.3)
Digital clubbing	20 (83.3)
Presence of wheezing	2 (8.3)
Pulse oximetry	93 ± 2.4
Sweat electrolyte technique	
Indirect technique	11 (45.8)
Gibson & Cooke technique	13 (54.1)
Sweat electrolyte value	103 ± 32.8
Sputum culture in the last trimester *n*:23	
Negative	10 (43,5)
*Pseudomona aeruginosa*	1 (4.34)
*Staphylococcus aureus*	7 (30,4)
Other microorganism	5 (21,7)
CF treatment	
Pancreatic enzymes	18 (75.0)
Vitamins	20 (83.3)
Nutritional supplement	17 (70.8)
Dornase alpha	19 (79.2)
Device for nebulized medication	24 (100)
7% Hypertonic saline solution	22 (91.6)
Inhaled antibiotic	1 (4.2)
Respiratory therapy	23 (95.8)
Clinical Outcomes	
Exacerbation in the last year	11 (45.8)
Average number of exacerbations in the last year *n*: 11	2.3 ± 2.6

BMI, body mass index; CT, computerized tomography; CF, cystic fibrosis.

Values as means ± standard deviation and *n* (%).

**Table 2:** Participants’ genetic study results.

Mutations in the CFTR gene	*n*:24
Homozygous p.F508del	8
Homozygous p.G542X	2
Heterozygous p.F508del and heterozygous c.489+1G>T p.(?)	1
Heterozygous p.F508del and heterozygous (c.3846G>A; p.(Trp1282Ter)	2
Homozygous c.1312A>G p.(Thr438Ala)	1
Heterozygous c.3368-2A>G p.(?) and heterozygous c.580-1G>T p.(?)	1
Heterozygous c.1000C>T p.(Arg334Trp) and heterozygous c.3484C>T p.(Arg1162Ter)	1
Homozygous p.V470M	1
Homozygous c.3484C>T p.(Arg1162Ter)	1
Negative genetic study	5
Without genetic study	1

CFTR, cystic fibrosis transmembrane conductance regulator.

The median absolute baseline FEV_1_ was 1,880 (1,355–2,325) ml and 98% (83%–110%) of predicted value and only 4/24 had bronchodilator responsiveness (FEV_1_ > 12%). No significant difference in FEV_1_% was observed between subjects with OSA and those without OSA (*P* = 0.56). Polysomnography was performed in 20 of 24 patients. The prevalence of obstructive sleep apnea was 66.7% in children younger than 13 years and 40% in children older than 13 years with a median total AHI of 4.2 (1.6–6.5), central AHI of 0.3 (0–1.7) and OAHI of 3.05 (1.2–5) (Table 3). Mean saturation during wakefulness was 90.2 ± 2.3, SpO2 during sleep was 89.4 ± 2.2, and desaturation index was 10.2 ± 4.6. A comparison was made between children with CF without SDB and children with SBD breathing. It was observed that patients with SDB exhibited a higher ODI (12.5 ± 4.0 vs. 6.1 ± 2.4). Regarding the echocardiographic variables, none of the patients had an intermediate or high probability of pulmonary hypertension, as the median pulmonary artery systolic pressure was 22 (20–27).

**Table 3:** Results of diagnostic tests performed on study participants.

Espirometry pre & post b2	*n*:23
FEV_1_ Pre ml	1,880 (1,355–2,325)
FEV_1_ Post ml	1,990 (1,390–2,610)
FEV_1_ (%) Pre	98 (83–110)
FEV_1_ (%) Post	97 (82–120)
FEV_1_ Post *n* (%)	
≤12%	19 (82,6)
>12%	4 (17,4)
FVC (%) Pre	0.83 (0.78–0.86)
FVC (%) Post	0.85 (0.80–0.90)
Echocardiogram	
PASP	22 (20–27)
TAPSE	21 (18–23)
Right ventricle acceleration	1 (4.2)
Polysomnogram *n*:20	
Total AHI	4.2 (1.6–6.5)
Central AHI	0.3 (0–1.7)
Obstructive AHI	3.05 (1.2–5)
Obstructive AHI ≥ 2	
≥ 13 years *n* = 5	2 (40,0)
≤ 12 years *n* = 15	10 (66,7)
T90	45.6 ± 37.5
T85	5.75 ± 13.9
T80	0.25 ± 0.8
SpO2 during wakefulness	90.2 ± 2.3
SpO2 during sleep	89.4 ± 2.2
Desaturation index	10.2 ± 4.6
Capnography	
≤35	7 (35.0)
≤40	3 (15.0)
≤45	9 (45.0)
≤50	1 (5.0)

FEV_1_, forced expiratory volumen in first second; ml, mililiters; FVC, forced vital capacity; PASP, pulmonary artery systolic pressure; TAPSE, tricuspid annular plane systolic excursion; AHI, apnea Hypopnea index; SpO2, pulse oximetry. Values as means ± standard deviation or median (p25–p75) and *n* (%).

**Figure 1:**
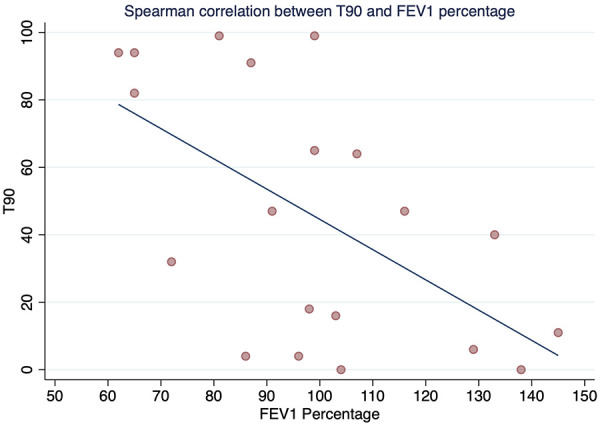
Spearman correlation between T90 and FEV1 percentage.

**Figure 2:**
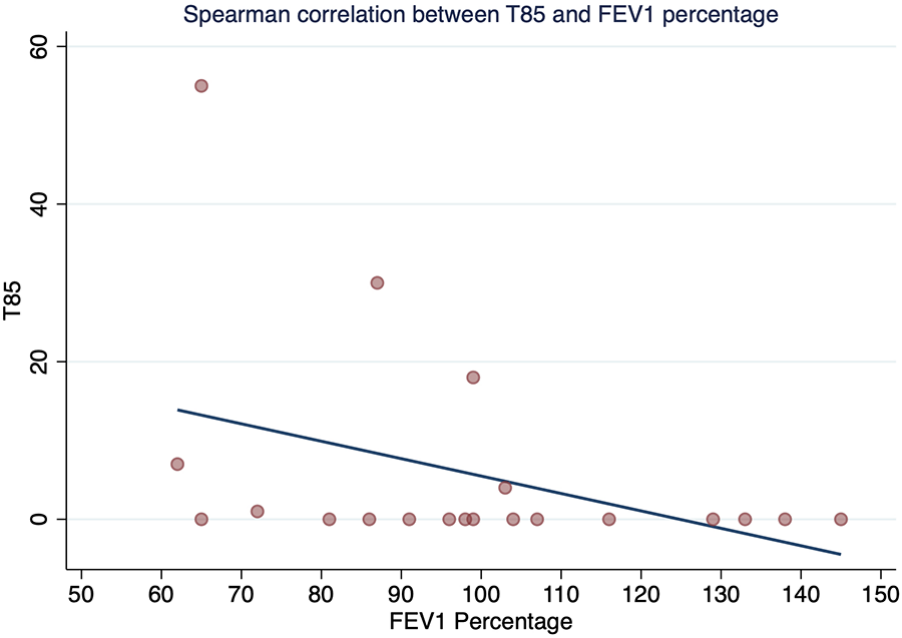
Spearman correlation between T85 and FEV1 percentage.

**Figure 3:**
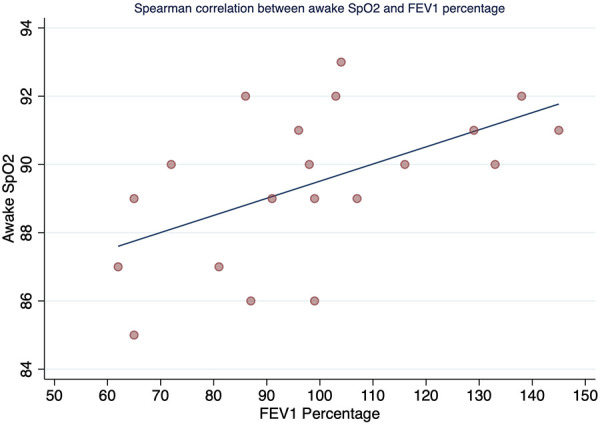
Spearman correlation between awake SpO_2_ and FEV1 percentage.

The Spearman correlation coefficient between FEV_1_ and T90 was rho −0.52 (*p*-value = 0.018) (Figure 1), and between FEV1 and T85 was statistically significant with rho −0.45 and *p*-value = 0.041 (Figure 2). A positive correlation was observed between FEV_1_ and SpO_2_ during sleep with rho 0.53 and a statistically significant value *p* = 0.014 (Figure 3), while between FEV_1_ and EtCO_2_ the rho was 0.44 with a *p*-value = 0.047.

## Discussion

This study showed that in this group of children aged 6 to 18 years with CF living at high altitude (2,640 m), there was a high prevalence of OSA (65%) with normal lung function, regardless of disease severity as determined by %FEV_1_. A moderate positive correlation was found between %FEV_1_ and SpO_2_ during sleep, and a moderate negative correlation was found between %FEV_1_ and T90 and T85, with a mean SpO_2_ during sleep of less than 90%. This suggests that a higher %FEV_1_ predicts a higher SpO_2_ during sleep, while a lower %FEV_1_ predicts a higher percentage of total sleep time with SpO_2_ less than 90% and less than 85% during sleep. Additionally, we observed a higher ODI in patients with CF and SDB compared to those with CF without SDB.

The results showed a high prevalence of OSA in children with CF compared to the prevalence in healthy children, ranging from 0.7%–13%, depending on the AHI cut-off points used to diagnose OSA (20, 21). The prevalence of OSA found in this study is similar to that reported in a meta-analysis conducted in children and adolescents with CF, which included six studies in which the pooled prevalence of OSA defined as AHI >1/h was 65% (95% CI: 0.54–0.76) and with OSA defined as AHI >2/h was 51.5% (95% CI: 0.18–0.84) (10). It is important to note that the criteria for OSA in our study are consistent with those established in the literature (22). It is possible that if a lower AHI cut-off had been used (e.g., AHI >1/h), the prevalence of OSA in these children with CF living at high altitude would have been higher, but the results could potentially have been affected by a higher frequency of false-negative cases, especially in children living at high altitude.

From a functional point of view, this group of children with CF did not have advanced or severe disease, with a normal FEV_1_ range, and no significant correlation was found between AHI and %FEV_1_. Like our study, Silva et al. reported that 87.9% of clinically stable children with normal or mildly impaired lung function had some type of sleep disorder, either respiratory or sleep architecture (23). As shown in our study and others, the severity of SDB in children with CF does not appear to be solely related to the degree of lung involvement as assessed by %FEV_1_ (24, 25). These findings highlight the need for early detection of SDB, before lung function deteriorates, to prevent the metabolic, cardiovascular, and neurocognitive effects of SDB (26), which increases the burden of the disease.

Although a high prevalence of OSA is recognized in children and adolescents with CF, the factors associated with its development and the role of living at high altitude are not fully understood. It is possible that nasal polyps and/or nasal mucosal inflammation, which occur in most patients with CF, may contribute to the presentation of OSA. However, we did not evaluate this aspect in our study (27). Regarding the effects of exposure to high altitude, it is likely that, as some authors have suggested in relation to children without CF, chronic adaptation to high altitude and an individual predisposition may lead to hyperventilation and a high frequency of micro-alerts, and consequently to a series of pathophysiological events culminating in obstructive apnea with even greater frequency and severity (28). However, studies in Andean populations living at high altitude have shown that chronic adaptation results in an increase in erythrocyte count with a subsequent increase in hemoglobin and a decreased hypoxic vasoconstrictor response. Regarding the increase in ventilation observed with acute exposure to high altitude, the increase in ventilation is not present in Andean natives living at high altitude and is similar to that of natives living at low altitude, both at rest and during exercise (29). However, the study of the normal values of arterial gases in our group shows lower values of PCO2 and PO2 in adults, which could indicate a degree of hyperventilation (7).

A positive correlation was found for %FEV_1_ (rho = 0.53, *p* = 0.014) with mean SpO_2_ during sleep, and a negative correlation was found between %FEV_1_ and T90 and T85. Comparison of these results with those of Uyan et al. (5) and Spicuzza et al. (30), These authors did not find a correlation between mean SpO_2_ during sleep and FEV_1_. In contrast, de Castro-Silva et al. (31) found a similar result to our study (correlation between mean SpO_2_ and FEV1; *p* < 0.001). These inconsistent results may be due to the different methodologies used, including the evaluation of different parameters, the use of different predictive equations to assess lung function, and differences in the CF severity profiles of the participants.

In the current study, patients had an average SpO_2_ during sleep of 89.4 ± 2.2 and a high ODI of 10.2 ± 4.6. Compared with results from studies conducted at sea level with similar functional characteristics (23, 31), mean SpO_2_ during sleep and SpO2 during wakefulness were lower and desaturation indices were higher. Thus, our data are consistent with the results of other studies regarding oxygen desaturation during sleep in patients with CF with a higher degree of severity (12, 32, 33). It is important to note that the patients in this study showed desaturation even when the disease was mild and they were in a period of clinical stability, suggesting the impact of chronic hypoxia in CF patients living at high altitudes.

An additional finding of our study was that none of the patients had an intermediate or high probability of pulmonary hypertension based on the pulmonary artery systolic pressure (PASP) value. Estimation of PASP is based on the tricuspid regurgitant velocity (TRV) using Bernoulli's equation. However, unlike in adults where the probability of pulmonary hypertension is determined from the TRV value (34), no studies have determined the normal values of PASP in children. Since there are currently no studies that have determined the probability of pulmonary hypertension from PASP, in consensus with the research group and taking into account the parameters used in other studies and reference values extrapolated from the adult population, the upper limit of normal (ULN) for right ventricular systolic pressure was set between 30 and 35 mmHg and >40 mmHg for the TTE diagnosis of pulmonary hypertension (35–38) considering that, according to the studies in adults, a PASP of <35 mmHg implies a TRV of less than 2.8, which confers a low probability of pulmonary hypertension (34, 39, 40). This finding is consistent with the results of another study by our group in children with OSA at high altitude, where none of the children had PASP levels above ULN, regardless of OSA severity (41).

Some limitations of this study include the fact that we did not have a control group of patients living at a lower altitude, and we did not evaluate the presence of nasal polyps, which may have contributed to the high prevalence of OSA. In addition to the small number of patients. Of the 24 patients enrolled in the study, 4 did not attend the polysomnogram, thus polysomnogram analysis was performed on only 20 of the 24 children enrolled. Furthermore, we have no data on the presence of polycythemia in our population and we have no data on the presence of hyperventilation during the sleep study. The strength of this study is that it is the first study to determine the impact of chronic altitude hypoxia in children with CF living at high altitude.

In conclusion, this study found a high prevalence of sleep apnea in children with CF living at high altitude - higher than the prevalence of sleep apnea in children without CF reported in the literature, but like children and adolescents with CF living at sea level. It also found an average desaturation during sleep higher than that found in children with CF with normal lung function living at sea level. In addition, this study found a negative correlation between FEV1 and T90 and T85 oxygenation indices and a positive correlation between FEV1 and SpO2 during sleep. Furthermore, this study identified a negative correlation between FEV1 and T90 and T85 oxygenation indices and a positive correlation between FEV1 and SpO2 during sleep. Additionally, this study found that those patients with CF and SDB exhibited a higher ODI, emphasizing the importance of early detection in this group of patients.

## Data Availability

The raw data supporting the conclusions of this article will be made available by the authors, without undue reservation.
